# Identification and analysis of oxygen responsive microRNAs in the root of wild tomato (*S. habrochaites*)

**DOI:** 10.1186/s12870-019-1698-x

**Published:** 2019-03-12

**Authors:** Yabing Hou, Fangling Jiang, Xiaolan Zheng, Zhen Wu

**Affiliations:** 1 College of Horticulture, Nanjing Agricultural University, Nanjing, 210095 China; 2 College of Horticulture, Henan Agricultural University, Zhengzhou, 450002 China

**Keywords:** Wild tomato, Hypoxia, Root, miRNA, miR171, miR390

## Abstract

**Background:**

MicroRNA (miRNA) are key players in regulating expression of target genes at post-transcriptional level. A number of miRNAs are implicated in modulating tolerance to various abiotic stresses. Waterlogging is an abiotic stress that deters plant growth and productivity by hypoxia. Dozens of reports mention about the miRNAs expressed in response to waterlogging and hypoxia. Despite the fact that tomato is a model vegetable but waterlogging sensitive crop, the role of miRNAs in hypoxia tolerance is poorly understood in tomato.

**Results:**

In this study, we investigated the differentially expressed miRNAs between hypoxia-treated and untreated wild tomato root by using high-throughput sequencing technology. A total of 33 known miRNAs were lowly expressed, whereas only 3 miRNAs showed higher expression in hypoxia-treated wild tomato root compared with untreated wild tomato root. Then two conserved and lowly expressed miRNAs, miR171 and miR390, were deactivated by Short Tandem Target Mimic (STTM) technology in Arabidopsis. As the results, the number and length of lateral roots were more in STTM171 and STTM390 transgenic lines compared with that of wild type plant, which partly phenocopy the increase root number and shortening the root length in hypoxia-treated wild tomato root.

**Conclusions:**

The differentially expressed miRNAs between hypoxia-treated wild tomato and control root, which contribute to the auxin homeostasis, morphologic change, and stress response, might result in reduction in the biomass and length of the root in hypoxiated conditions.

**Electronic supplementary material:**

The online version of this article (10.1186/s12870-019-1698-x) contains supplementary material, which is available to authorized users.

## Background

Tomato is an important vegetable crop and it is highly sensitive to waterlogging [1]. As an important abiotic stress, waterlogging mainly negatively affects plant growth and productivity by oxygen deprivation [2]. It has been reported that reduction of gas exchange is the primary consequence of soil waterlogging, which may result from the relatively slow diffusion of gases, including oxygen, in water [2]. Two typical tomato responses to waterlogging are epinasty and adventitious root formation. Multiple evidences have shown that adventitious root production in waterlogged tomato plants is mediated by the crosstalk of ethylene and auxin signaling [3]. The regulation of plant response to low oxygen involves various transcriptional factors, which act in both metabolic reprogramming and morphological adaptations [4, 5].

Plant microRNAs (miRNAs) are 18–24 nucleotides (nt) long noncoding small RNAs. Generally, miRNAs negatively regulate their target genes expression by binding to mRNA sequences, leading to mRNA cleavage or translation repression [6]. Recently, miRNAs were also reported to regulate gene expression by controlling gene methylation [7]. Increasing evidences suggested that miRNAs are indispensable in controlling multiple biological processes including plant development, hormone homeostasis, and response to environmental stimuli [8–11]. Altering the expression of one or more miRNAs can result in multiple visible morphological defects in plants [12–14]. In addition, majority of the miRNA targets are transcriptional factors (TFs) [15] . Combined with the fact that multiple TFs responded differentially through time and oxygen concentration-dependent manner [4, 5], miRNAs may also be involved in determining the morphological changes of plant growth under low-oxygen conditions.

Dozens of studies have been done upon waterflooding and hypoxia-responsive miRNAs. A previous study indicated that 46 miRNAs from 19 families and three tasiRNA families were differentially expressed in Arabidopsis after 5 h of hypoxia treatment [16]. However, pri-miR159c was the most induced after 30 min, and mature miR391 showed the modest increase after 4 h in hypoxiated Arabidopsis [5]. miR159, miR164, miR167, miR393, miR408, and miR528 are mainly involved in root development and stress responses, but also found to be key regulators under short-term waterlogging conditions in three maize inbred lines [17]. Comparison of the 32 waterlogging-responsive miRNAs’ expression in both waterlogged and control crown roots showed that most of them were consistently down-regulated under 1–3 days waterlogging in the two maize inbred lines [18]. Ren et al. reported that seven conserved and five novel miRNAs were differentially expressed in response to flooding stress in Populus [19]. However, there is little information about the miRNAs involved in tolerance to long-term oxygen deprivation in the root of tomato.

In this study, high-throughput sequencing technology was employed to detect the differentially expressed miRNAs between hypoxiated and control wild tomato roots. The results showed that a total of 33 known miRNAs were lowly expressed, while 3 known miRNAs were highly expressed in hypoxia-treated wild tomato root compared with that of the control plant. Subsequently, to phenocopy the shorter but more roots phenotype of hypoxia-treated wild tomato root, two lowly expressed conserved miRNAs, miR171 and miR390, were blocked by using short tandem target mimic (STTM171 and STTM390) in Arabidopsis. As expected, more and longer roots were observed in STTM171 and STTM390 transgenic lines. All these results indicated that the phenotype of hypoxia-treated wild tomato root may result from the differentially expressed miRNAs.

## Methods

### Plant material and growth conditions

Hypoxia sensitive wild tomato *S. habrochaites* 178 (Accession No. LA1777, Tomato Genetics Resource Center (TGRC)) and cultivar tomato *S. lycopersicum* Fenzhenzhu (Henan Yuyi Seed Industry Technology Co., Ltd., Zhengzhou, China) were used as the material and cultivated under hydroponics. For Arabidopsis, Columbia-0 (Col-0) was used as the wild type (WT), all plants were grown in long day condition with 16 h light/8 h dark at 23 °C in an incubator.

### Hypoxia treatment and morphometry

The seeds of tomato were sowed on the solid medium (vermiculite: peat: pearlite = 2:1:1) after germination. 27 days old seedlings (counted from sowing) were transferred to plastic pot (65 cm × 40 cm × 15 cm) covered by a PVC plastic board, which was drilled holes with the spacing of 7 cm × 7 cm. The seedlings were then put into the 1/2 Hoagland liquid media with 6.1 ± 0.2 pH and 2.2–2.5 ms cm^− 1^ electrical conductivity for two days prior to treatment. After that, two treatments, hypoxia and control at rhizosphere, were conducted. The treatments followed the protocols of Gasch et al., [20]. Specifically, half Hoagland solution in each pot was aerated by an air pump, which was controlled by a dissolved oxygen control instrument (YiTang (China) co., LTD, Haerbin, China) to maintain the dissolved oxygen content at 0.5–2.0 mg L^− 1^ and 7.0–8.0 mg L^− 1^ for hypoxia and control at rhizosphere, respectively. For hypoxia at rhizosphere, N_2_ was used as source of the air pump, while for the control plant (CK), air was used as a source of the air pump. The liquid media was replaced every week. The materials were collected at 12 days after treatment. Within each treatment, half of roots from 10 plants were placed in liquid nitrogen and stored at − 80 °C for RNA extraction, and half were collected and used to measure the phenotype.

Regarding to morphological index measurement, root number, length and diameter, dry weight of root and shoot were assayed with 6 repeats. In detail, root number was counted manually, root diameter and length were respectively measured by vernier caliper and ruler, dry weight of root and shoot were determined by electronic scales after the samples thoroughly dried in drying oven. In addition, the data of root number and diameter were showed by the average, while root length and dry weight of root and shoot were presented by the average of per plant.

### RNA extraction and quantitative real-time PCR (qRT-PCR)

To detect the expression of miRNAs in hypoxia-treated and control plant. Total RNA of hypoxia-treated and control plant roots was isolated by Trizol (Invitrogen) following the manufacturer’s instructions. Stem-loop qRT-PCR was used to measure the targeted miRNA expressional level, and was followed the procedure of Peng et al. [21]. In brief, 1 μg of total RNAs was reverse-transcribed with the help of reverse transcriptase (Promega) and miRNA RT-primers. 5 μl of the 1:20 diluted cDNA was used as template in a 20 μl PCR system, mixed with the 10 μl SYBR green reaction solution (SYBR® Green QRT-PCR Master Mix; Toyobo), 1 μl forward prime, 1 μl Stem-loop_U, and 3 μl ddH2O. The qPCR system was firstly pre-incubated at 95 °C for 5 min, and then went to the 40 cycles including denaturation at 95 °C for 15 s, annealing at 60 °C for 15 s, and extension at 72 °C for 32 s by using BioRad iQ5 sequence detection system (BioRad, USA). Standard qRT-PCR was carried out to detect miRNA target gene’s expression. The same batch of Trizol isolated total RNA was used to yield cDNA (High-Capacity cDNA Archive Kit, Applied Biosystems). qRT-PCR was performed with an Applied Biosystems step one instrument using the SYBR Green PCR master mix kit (Applied Biosystems) according to the manufacturer’s instructions with U6 mRNA as an internal control described by Huang et al. [12]. The sequences of primers were listed in Additional file 1. Values were obtained by normalizing to U6 and actin then comparing the normalized values to the control plants, respectively. The relative levels of gene expression were calculated using the 2^-ΔΔCT^ method.

### Small RNA deep sequencing and differential expression analysis

Total RNAs were extracted from the roots of hypoxia-treated and the control plants for deep sequencing. Small RNA sequencing and data analyses were performed as described previously [21]. Briefly, small RNAs were firstly isolated by polyacrylamide/urea gel electrophoresis, then 3′ Solexa DNA adaptor was ligated to the purified small RNAs, followed by 5′ Solexa RNA adaptor ligated. Purified small RNAs product was then reverse transcribed and amplified by PCR. The resulting small RNA library was submitted to Genome Analyzer (Illumina) to sequence according to the manufacturer’s protocol. After removing the adapter and low-quality sequences from the raw data product from small RNA sequencing, the clean reads were mapped to the tomato miRNA precursor deposited in miRBase 21 directly.

To compare with the abundance of known miRNAs, only the tags meet with the following criteria were defined as know miRNAs [22]: (1) sequences can be perfectly mapped onto tomato miRNA precursors deposited in miRBase; (2) the start position of the tag must be between + 2 and − 2 nt away from the 5′ end of the mature miRNA on the precursor. The expression of specific miRNAs was calculated by the abundance of this miRNA detected in the library. To compare the difference of the same miRNA in the two libraries, the miRNA’s expression was normalized to the total count of clean reads.

(I) Normalization formula:

$$ \mathrm{Normalized}\ \mathrm{expression}=\mathrm{actual}\ \mathrm{miRNA}\ \mathrm{count}/\mathrm{total}\ \mathrm{count}\ \mathrm{of}\ \mathrm{clean}\ \mathrm{reads}\times \mathrm{1,000,000}. $$

After normalization, we set the expression to 0.01 for miRNAs that were not expressed in one of the libraries. In addition, the miRNA whose expression was lower than 1 transcript per million (TPM) in both the hypoxia-treated and control library were filtered, and the remainder miRNAs were used for differential expression analysis.

(II) Calculate the fold-change and *P*-value from the normalized expression.

Fold-change formula:

$$ \mathrm{Fold}-\mathrm{change}={\log}_2\ \left(\mathrm{normalized}\ \mathrm{expression}\ \mathrm{of}\ \mathrm{miRNA}\ \mathrm{in}\ \mathrm{control}/\mathrm{normalized}\ \mathrm{expression}\ \mathrm{of}\ \mathrm{miRNA}\ \mathrm{in}\ \mathrm{hypoxia}-\mathrm{treated}\ \mathrm{root}\right). $$

*P*-value formula:

$$ p\left(y\left|x\right.\right)=\left(\frac{N_2}{N_1}\right)\frac{\left(x+y\right)!}{x!y!{\left(1+\frac{N_2}{N_1}\right)}^{\left(x+y+1\right)}}\kern0.5em {\displaystyle \begin{array}{c}C\left(y\le {y}_{\mathrm{min}}\left|x\right.\right)=\sum \limits_{y=0}^{y\le {y}_{\mathrm{min}}}p\left(y\left|x\right.\right)\\ {}C\left(y\ge {y}_{\mathrm{max}}\left|x\right.\right)=\sum \limits_{y\ge {y}_{\mathrm{min}}}^{\infty }p\left(y\left|x\right.\right)\end{array}} $$

where N_1_ is the total number of clean tags in hypoxia-treated root, x is the number of miRNAs surveyed, N_2_ is the total number of clean tags in control, and y is the number of homologous miRNAs in hypoxia-treated root.

If the |fold-change| of the miRNA is ≥0.6, simultaneously 0.01 ≤ *P*-value < 0.05, represents this miRNA differentially expressed, while a P-value < 0.01 indicates that the miRNA was significantly different between hypoxia-treated and control wild tomato root.

### Identification of novel miRNAs, prediction and validation of miRNA targets

To identify novel miRNAs, the following analyses were carried out as described by Peng et al. [23]. In briefly, small RNAs that might be generated from rRNA, tRNA, small nuclear RNA (snRNA), small nucleolar RNA (snoRNA), and siRNA were removed, and small RNAs mapped to repetitive and exon regions were filtered out as well. Then, the remainder were subjected to ‘MIREAP’ (https://sourceforge.net/projects/mireap/) to identify the novel miRNA candidates. Finally, the criteria proposed by Meyers et al. [24] were used to identify the novel miRNAs. To increase the reliability, the potential miRNAs who have their corresponding miRNA* sequences present, or whose abundance have more than 100 TPM in at least one of the two libraries, were finally defined as novel miRNAs.

The potential miRNA targets were predicted and validated using psRNATarget [25] and DPMIND [26] with default parameters, respectively. The sequences of different expressed known miRNAs were used as custom sequences. The *Solanum lycopersicum* (tomato), transcript, cDNA library, version 2.4 was used as the genomic library for the target search.

### Vector construction and transformation

To study the function of differentially expressed miRNAs identified from hypoxia at rhizosphere, a popular technique, short tandem target mimic (STTM), was employed to block miR171 and miR390’s expression in Arabidopsis. STTM vectors construct were generated by the following procedures described by Tang et al. [13]. In brief, specific STTM primers were designed and synthesized. PCR was carried out by using LongAmp, dNTPs, and LongAmp buffer from New England BioLabsInc (NEB). STTM fragment was inserted into the pOT2-Poly-Cis vector firstly by PCR, then through SwaI digestion (NEB), and lastly via T4 DNA ligase (NEB) catalyzed ligation. The STTM containing pOT2-STTM vector was amplified in DH5α competent cells. We removed the replication origin from pOT2-STTM by origin deletion PCR. The origin deleted pOT2-STTM together with pFGC5941-PacI binary vector was incubated with PacI endonuclease (NEB) and then were ligated together by T4 DNA ligase (NEB). The STTM-pFGC5941 plasmid was amplified in DH5α competent cells. All the prepared STTM-pFGC5941 plasmids for different miRNAs were sent to Sangon Biotech (Shanghai) Co., Ltd. for sequencing. The ones with exactly correct sequence were used to transform the plants. Transgenic Arabidopsis lines were selected by spraying 0.1% glufosinate (bar herbicide).

### IAA content measurement

Contents of IAA was assayed by high-performance liquid chromatography-tandem mass spectrometry (ESI-HPLC-MS/MS) as previously reported [27]. Briefly, 0.2 g samples from fresh roots of hypoxia-treated and control plants were collected and grounded into powder using liquid nitrogen. 3 ml isopropanol / hydrochloric acid extraction buffer was added first and followed by shaking at 4 °C for 30 min, after that, 5 ml dichloromethane was added and shaken at 4 °C for 30 min. Then organic phase was dried by nitrogen protected from light and dissolved by 400 μl methanol with 0.1% formic acid. HPLC-MS/MS analysis was conducted in ZOONBIO BIOTECHNOLOGY (Nanjing, China) after filtering with 0.2 μm filter membrane.

## Result

### Hypoxia reduced plant size and root length, but increased the root number in tomato

**Fig. 1:**
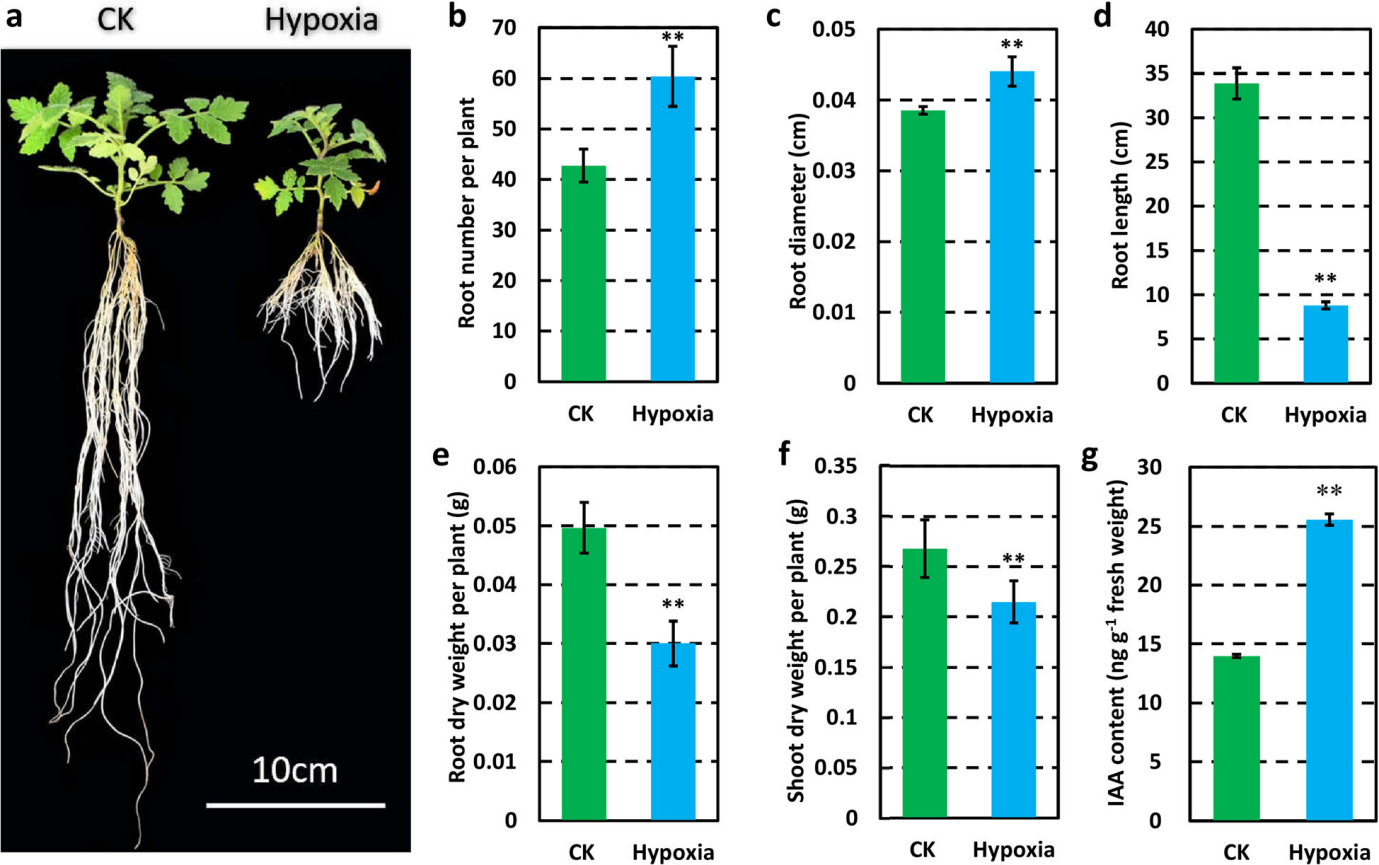
Phenotype of 41 days old wild tomato plant under 12 days hypoxia treatment at root rhizosphere and the control plant. **a** The whole plant morphology of hypoxia-treated wild tomato and the control plant (CK). Scale bars, 10 cm. Statistical analysis of root number (**b**), root diameter (**c**), root length (**d**), dry weight of root (**e**), dry weight of shoot (**f**), and IAA content (**g**). Asterisks indicate statistically significant differences compared with control by Student’s t test (*P < 0.05; **P < 0.01)

Hypoxia-treated plants showed smaller size in wild tomato plant (Fig. 1a). Compared with control plant, hypoxia-treated plants, most conspicuously showed increased root number (Fig. 1a, b), root diameter (Fig. 1c), and reduced root length (Fig. 1a, d), dry weight of root (Fig. 1e), and dry weight of shoot (Fig. 1f). Statistical data showed that the increase in the values of root number and root diameter were 41.36% (*P* < 0.01) and 14.20% (P < 0.01), respectively. And the decreased range of root length, dry weight of root, and dry weight of shoot were 285.77% (P < 0.01), 65.35% (P < 0.01), and 24.69% (P < 0.01), respectively. On the contrary, higher auxin content was presented in hypoxia-treated wild tomato root (Fig. 1g). Consequently, these results suggested that hypoxia-treated root rhizosphere significantly affected multiple traits, especially for roots in wild tomato. In addition, similar phenotypes were observed in cultivar tomato, Fenzhenzhu as well (Additional file 2).

### Differential expressions of overall small RNAs between hypoxia and control plant

**Fig. 2:**
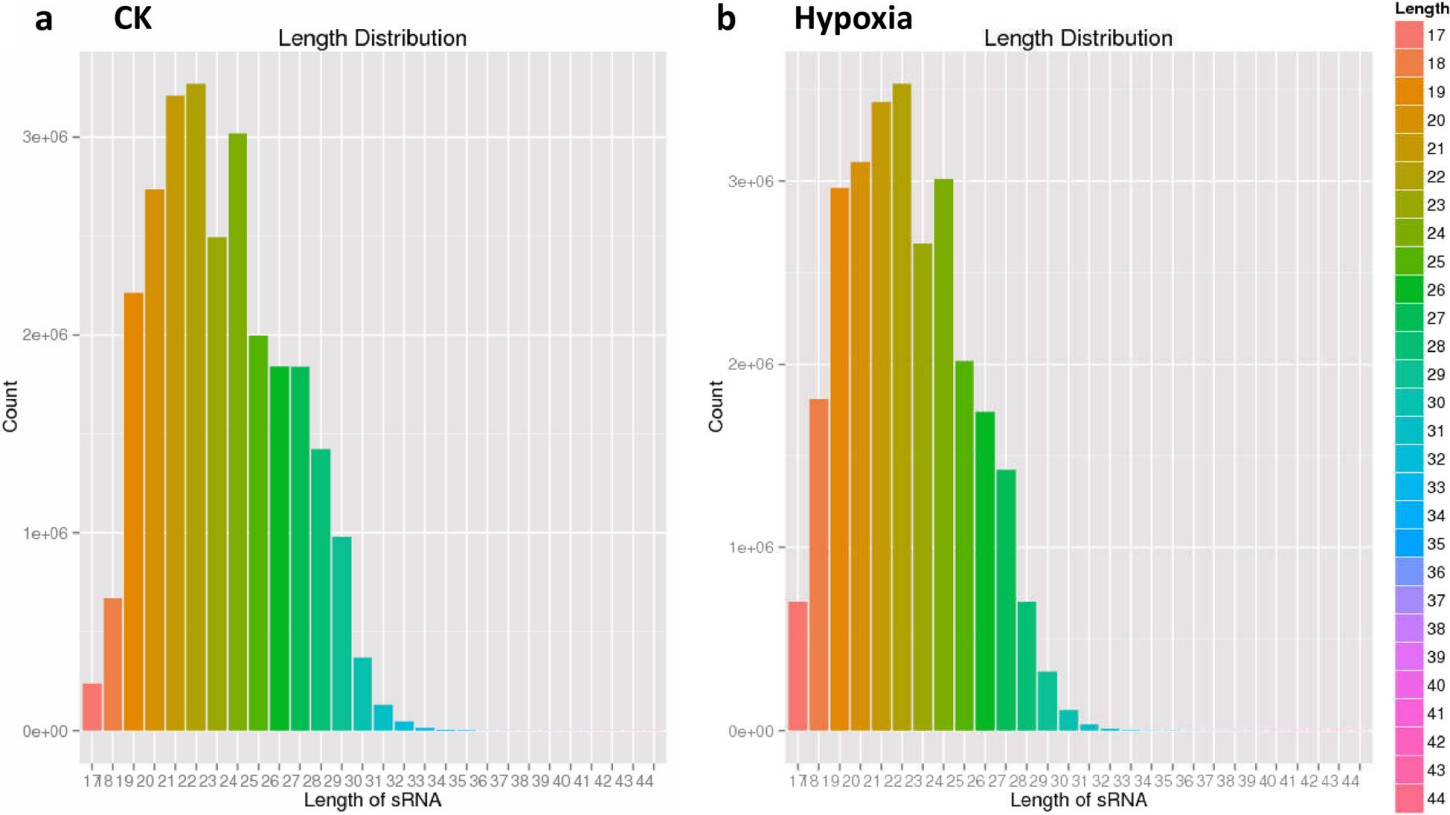
Summary of the length distribution of reads from control (**a**) and hypoxia-treated (**b**) wild tomato root produced by high-throughput sequencing technology

In order to detect the differentially expressed small RNAs between hypoxia and control wild tomato, high-throughput sequencing technology was employed, using the small RNA libraries made from the roots of both the samples. The roots from hypoxia and control wild tomato at 12 days after the hypoxia treatment were used to isolate small RNAs for sequencing. After trimming adaptor sequences and removing those reads with low quality and lengths smaller than 17 nucleotides, about 26,260,294 and 26,874,882 high-quality small RNA reads were obtained from control and hypoxia-treated wild tomato roots, respectively. In total, 9,731,252 and 10,476,168 reads, representing 37.06 and 38.98% of total clean reads, were perfectly matched to the tomato genome by analysis using SOAP [28] (Table 1). In addition, small RNA detected by high-throughput sequencing covered almost every kind of RNA, including miRNA, rRNA, sRNA, tRNA, snoRNA, snRNA, repeat associated sRNA, and degradation tags of exons or introns with various abundance (Table 1). In general, two major small RNA populations were found to be abundant according to their lengths in plant, one is 21 nt, the other is 24 nt. In our datasets, among millions of high-quality small RNAs from these two libraries, 19–24 nt small RNAs were predominant in both hypoxia-treated and control wild tomato roots (Fig. 2a and b), which may result from species and tissue-specific expressions of small RNAs.

**Table 1:** Distribution of small RNAs in different categories

Category	CK		Hypoxia	
	Reads	Frequency (%)	Reads	Frequency (%)
Clean	26,260,294	100	26,874,882	100
un_mapped^$^	16,529,042	62.94	16,398,714	61.02
Repeat	4,170,780	15.88	4,981,128	18.53
Intergenic	2,849,292	10.85	3,137,662	11.68
miRNA	643,669	2.45	389,909	1.45
rRNA	675,383	2.57	443,956	1.65
Exon	669,852	2.55	721,513	2.68
Intron	670,160	2.55	759,226	2.83
sRNA	33,612	0.13	26,651	0.1
Precursor	5962	0.02	7150	0.03
tRNA	5067	0.02	2239	0.01
snoRNA	4327	0.02	3148	0.01
snRNA	3148	0.01	3586	0.01

^$^The sequences not mapping to the genome of tomato

### Majority of the miRNAs were expressed lowly in hypoxia-treated wild tomato roots

**Fig. 3:**
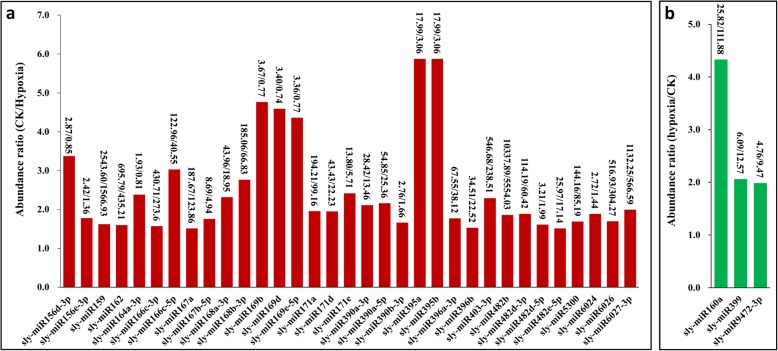
Comparison of the differential expression of known miRNAs between hypoxia and control wild tomato roots. Down (**a**) and up (**b**) regulated miRNAs in the roots of wild tomato treated by hypoxia at root rhizosphere. Abundance ratio of the normalized differentially expressed known miRNAs with an abundance of at least 1 TPM in one of the datasets are presented. The normalized miRNA abundance of each miRNA is shown on top of the bars, with numerator and denominator representing the normalized miRNA abundance in control and hypoxia-treated wild tomato roots, respectively

**Fig. 4:**
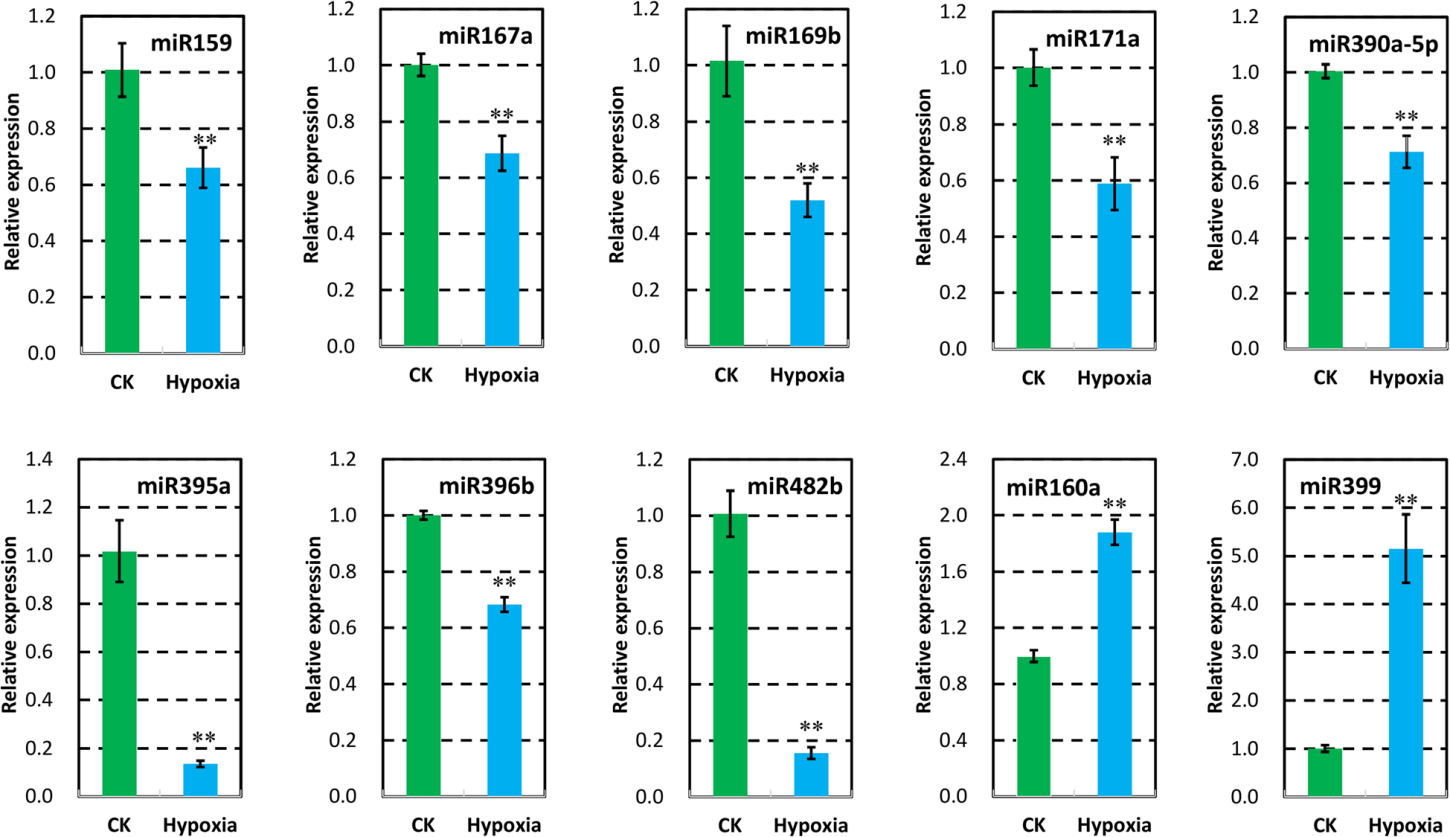
The expression of differentially expressed miRNAs verified by stem-loop qRT-PCR. Except miR160a and miR399, all others are down-regulated in hypoxia-treated wild tomato compared to control wild tomato roots (CK). U6 served as internal control. Asterisks indicate statistically significant differences compared with control by Student’s t test (*P < 0.05; **P < 0.01)

In total, 101 and 96 known miRNAs were obtained by the Solexa sequencing method from control and hypoxia-treated wild tomato roots, respectively (Additional file 3). Interestingly, differential expression analysis showed that 36 known miRNAs were more than 1.5 times lowly or highly expressed between the two samples, and 33 miRNAs, which accounted for 91.67% of these miRNAs, were down-regulated in hypoxia-treated wild tomato root (Fig. 3, Additional file 4). In short, compared with control, the expression abundance of two members of miR395 showed 5.88-fold decrease and represented largest reduction in hypoxia-treated wild tomato roots. The abundance of miR482b, miR159, miR6027-3p, miR162, miR403-3p, and miR166c-3p were higher than 400 TPM (transcripts per million) in one of our datasets and showed lower expression in hypoxia-treated wild tomato root. In addition, 25 other miRNAs showed at least a 1.5-fold decrease in hypoxia-treated wild tomato root, with the highest abundance ratio of 4.77 to 1 observed for miR169b (Fig. 3a, Additional file 4). However, miR160b, miR399, and miR472-3p had 1.5-fold up regulated in hypoxia-treated wild tomato root compared with control plant (Fig. 3b, Additional file 4). To further check the reliability of high-throughput sequencing, qRT-PCR was performed to study the transcripts of 10 differentially expressed known miRNAs between hypoxia and control wild tomato root obtained from high-throughput sequencing (Fig. 4, Additional file 5). The results showed that the tested miRNAs were preferentially expressed in control wild tomato root, which was consistent with the result of high-throughput sequencing.

### Expression patterns of differentially expressed known miRNAs targets

**Fig. 5:**
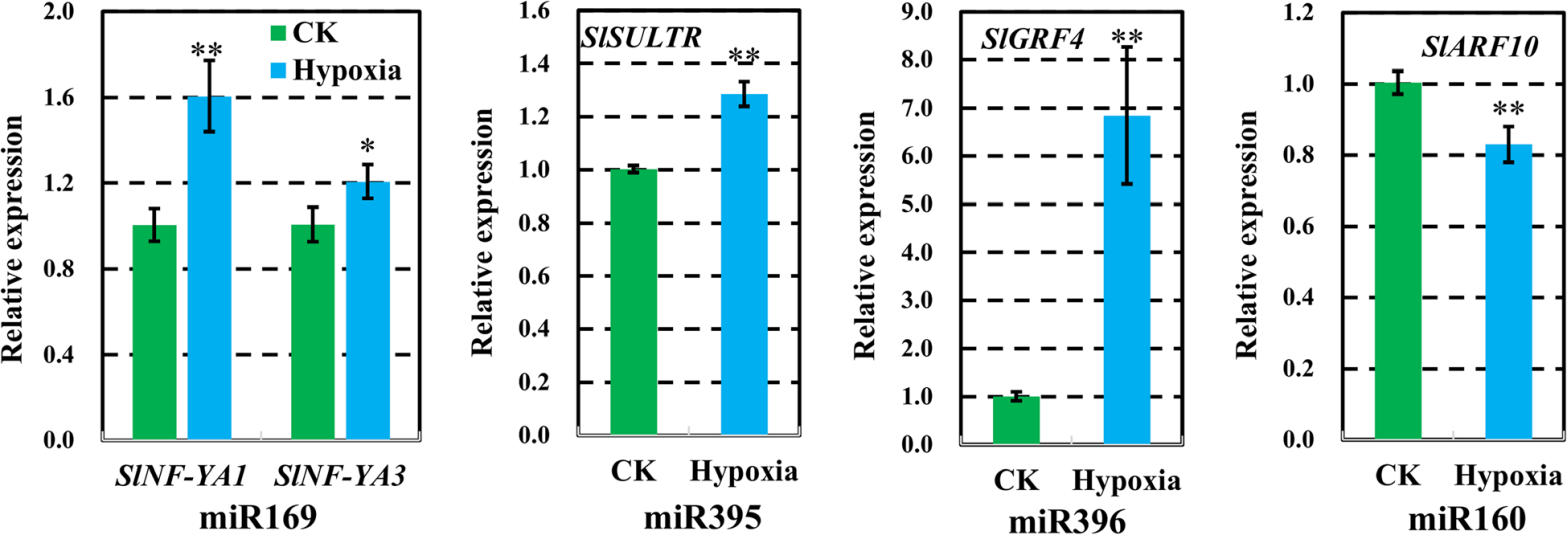
The expression of differentially expressed miRNA targets detected by qRT-PCR. The targets show inverse expression profiles compared to their targeting miRNAs. U6 served as internal control. Asterisks indicate statistically significant differences compared with control by Student’s t test (*P < 0.05; **P < 0.01)

It has been reported that the targets of the conserved miRNAs between Arabidopsis and rice, transcription factors account for approximately 70% of all the identified targets [29], indicating that miRNAs may act as important factors in regulating plant development. To analyze the function of these differentially expressed miRNAs between control and hypoxia-treated wild tomato root, their targets were predicted and validated using the psRNATarget [25] and DPMIND [26]. As a result, 575 target mRNAs were found for the 36 differentially expressed known miRNAs by psRNATarget, while 61 target mRNAs were validated as the target of the 23 differentially expressed known miRNAs by DPMIND (Additional files 6 and 7). In addition, the expression of 5 mRNAs targeted by 4 miRNAs: miR169, miR395, miR396, and miR160, were assayed by qRT-PCR. The results indicated that all these targets except *SlARF10* showed higher expression in hypoxia-treated wild tomato root, which is consistent with the fact that the targets are always negatively regulated by their corresponding miRNAs (Fig. 5, Additional file 8).

### Two highly expressed novel miRNAs were identified in the root of wild tomato

**Fig. 6:**
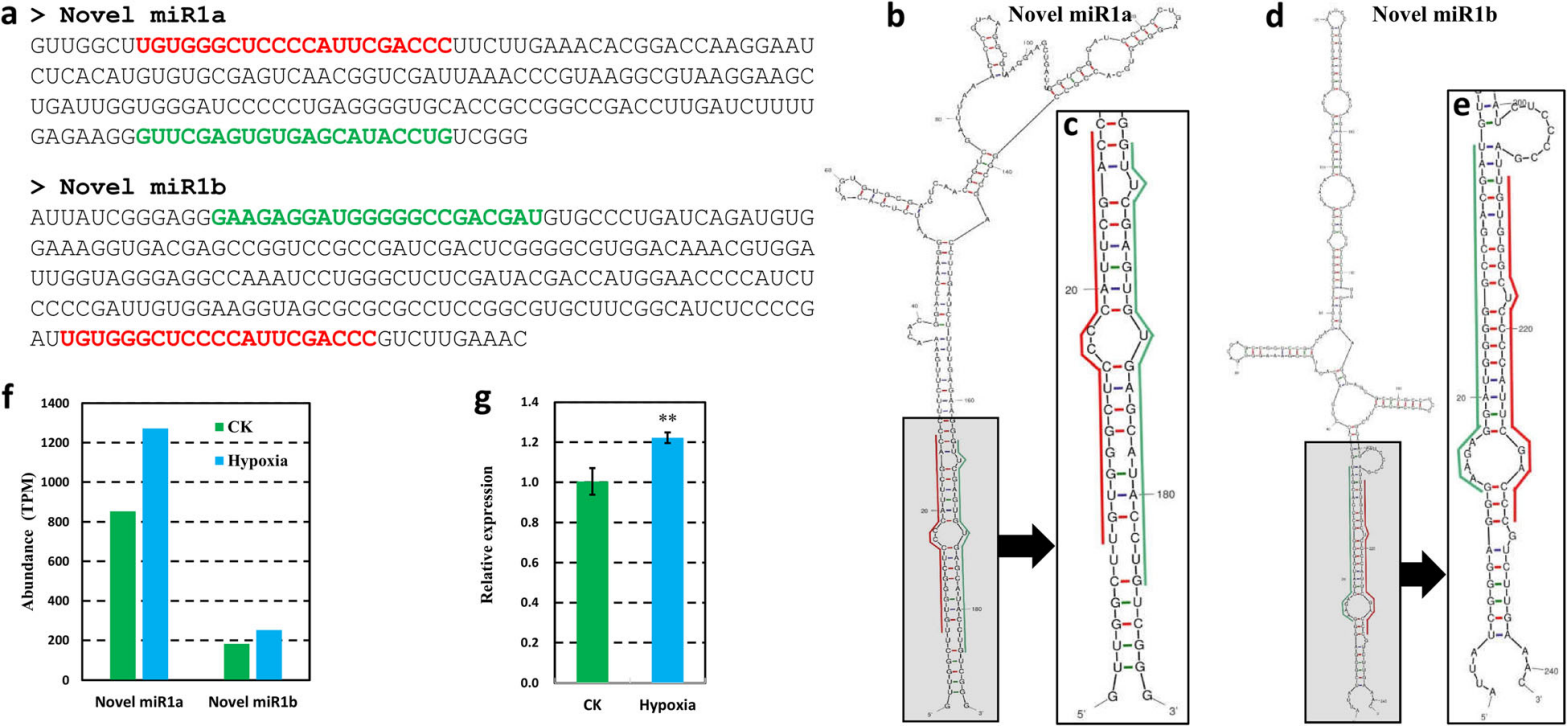
Novel miRNAs identified in the root of wild tomato. **a** The precursors of novel miRNAs identified in this study. **b-d** The stem-loop secondary structures of the novel miRNAs predicted by mfold (http://unafold.rna.albany.edu/?q=mfold) (**c**) and (**d**) are the zoomed regions of novel miRNAs and their corresponding miRNA*s. The nucleotides marked by the red line refer to the miRNA, whereas the nucleotides marked by the green line refer to the miRNA*. **f** The expression abundance of novel miRNAs identified in this study. **g** The relative expression of novel miRNAs in hypoxia-treated and control wild tomato root by stem-loop qRT-PCR. The two novel miRNAs were detected at the same time as they share the same mature miRNA sequence. Asterisks indicate statistically significant differences compared with control by Student’s t test (*P < 0.05; **P < 0.01)

To identify novel miRNAs in the root of wild tomato based on the small RNA-seq datasets, we looked for unreported miRNAs candidates collected from MIREAP and selected those candidates whose corresponding miRNA*s were also identified in our datasets [24]. Following these criteria, 2 novel miRNAs generated from the different loci of the genome, but showed the same sequence, and belonged to one family, were identified to have perfect stem-loop secondary structures (Fig. 6). Both of the novel miRNAs, novel miR1 and novel miR2, were observed to be highly expressed (>100TPM) in both of our datasets with slightly higher expression in hypoxia-treated wild tomato root compared with that of control plant root. For instance, the normalized abundance of novel miR1 were 1272.13 and 854.24 TPM in hypoxia-treated and control wild tomato root, respectively, and showed a 1.49-fold increase in hypoxia-treated wild tomato root (Fig. 6f). Furthermore, the expressions of these two newly identified miRNAs were validated by stem-loop qRT-PCR, and the results were consistent with the data we detected from high-throughput sequencing (Fig. 6g). To better investigate the function of the novel miRNAs, the target mRNAs were predicated and validated using psRNATarget [25] and DPMIND [26], respectively. As the result, only 2 target mRNAs were found as the targets of these two novel miRNAs (Additional file 9), whereas no corresponding target was verified by degradome sequencing based database.

### Lower expression of miR171 and miR390 may partly contribute to the phenotype of hypoxia-treated wild tomato root

**Fig. 7:**
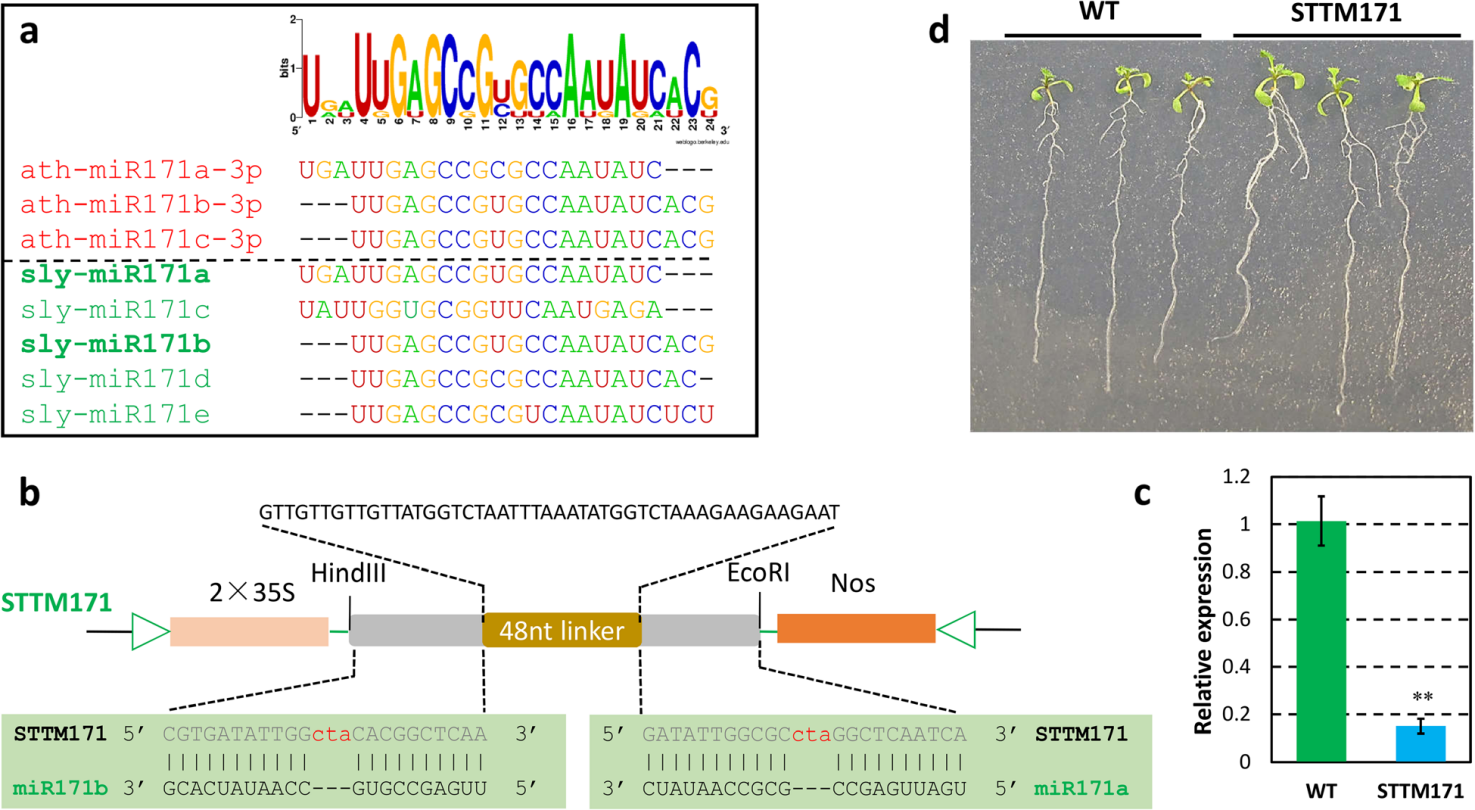
More and longer lateral roots presented in Arabidopsis STTM171 transgenic lines. **a** Sequence alignment of members of miR171 family of Arabidopsis and tomato. The name marked in bold was the miRNA used to construct STTM171. **b** Schema chart of STTM171 vector construction. **c** Comparison of the expression of miR171b in STTM171 transgenic plant and wild type (WT). Asterisks indicate statistically significant differences compared with WT plants by Student’s t test (*P < 0.05; **P < 0.01). **d** The phenotypes of two weeks old seedling of representative STTM171 transgenic plants compared with the WT plants

**Fig. 8:**
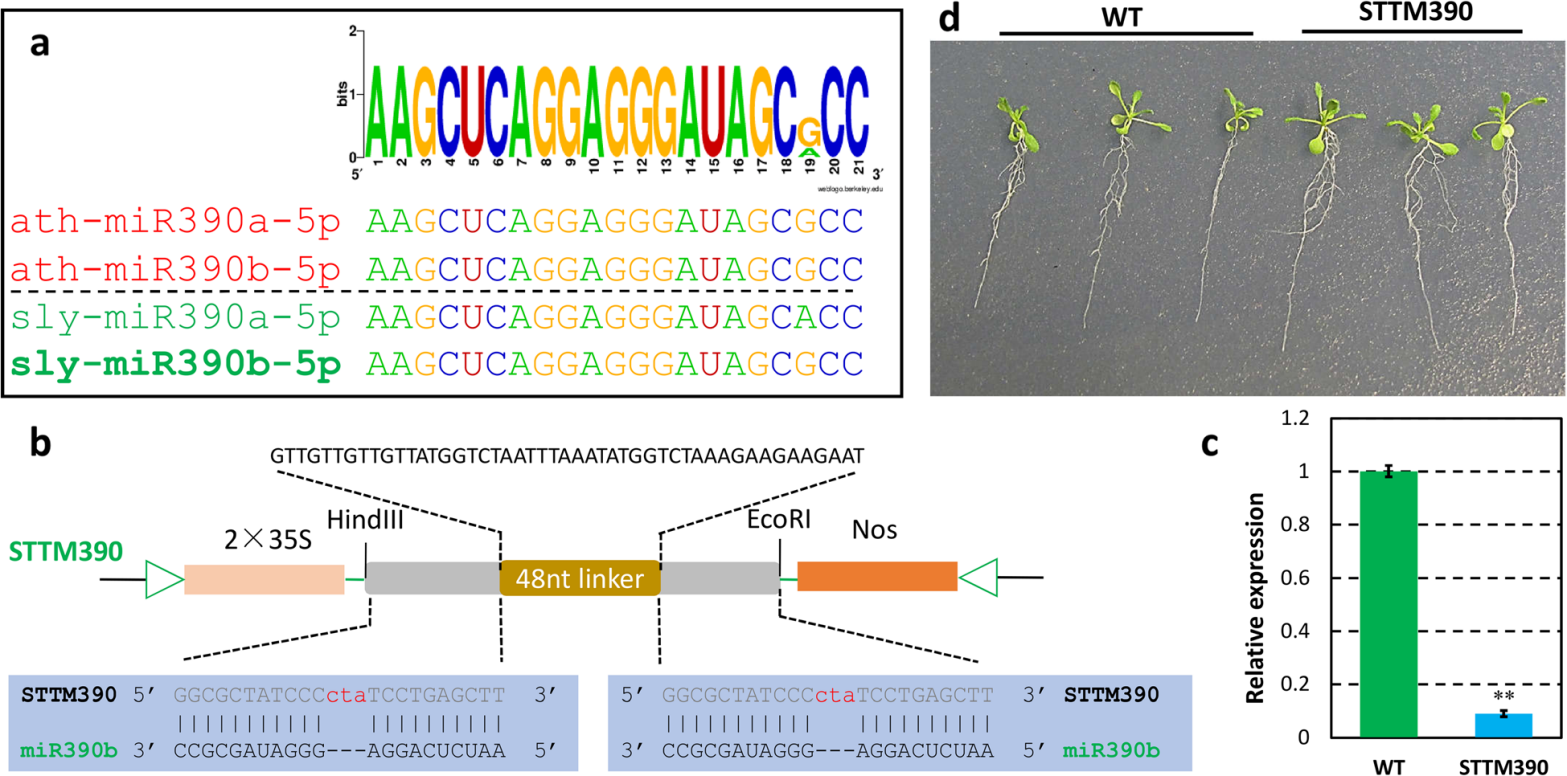
More and longer lateral roots presented in Arabidopsis STTM390 transgenic lines. **a** Sequence alignment of members of miR390 family of Arabidopsis and tomato. The name marked in bold was the miRNA used to construct STTM vector. **b** Schema chart of STTM390 vector construction. **c** Comparison of the expression of miR390b-5p in STTM390 transgenic plant and wild type (WT). Asterisks indicate statistically significant differences compared with WT plants by Student’s t test (*P < 0.05; **P < 0.01). **d** The phenotypes of two weeks old seedling of representative STTM390 transgenic plants compared with the WT plants

To further check whether these differentially expressed miRNAs actually contribute to the phenotypes present in hypoxia-treated wild tomato roots, STTM technology [13] was applied to suppress those miRNAs, which were found to be down-regulated in hypoxia-treated wild tomato roots. Two conserved miRNAs between tomato and Arabidopsis, miR171 and miR390, were selected and the two miRNA blocking lines were marked as STTM 171 and STTM390, respectively (Figs. 7a, b and 8a, b). As expected, the expression of the miR171 and miR390 were dramatically reduced in the STTM171 and STTM390 transgenic lines (Figs. 7c and 8c). Compared with two weeks old wild type (WT) plant, more and longer lateral roots were present in STTM171 and STTM390 transgenic plants (Figs. 7d and 8d, and Additional file 10). However, no significant changes in primary root length were observed in STTM171 and STTM390 transgenic plants compared with WT (Figs. 7d and 8d). All these results indicated that more roots of hypoxia-treated wild tomato plants may partly result from the lower expression of miR171 and miR390 in the root of hypoxia-treated wild tomato compared with that of control plant.

## Discussion

Although increasing number of studies about miRNAs and their expressions under hypoxia and waterlogging have been reported, there are fewer studies focused upon the plant growth in long-term oxygen deprivation of the root, especially for the model vegetable crop, tomato. In this study, two small RNA libraries from wild tomato roots of hypoxia-treated and control plants were generated. As the results, among the miRNAs with altered expression, a total of 33 known miRNAs were found to be lowly expressed, while 3 known miRNAs were highly expressed in hypoxia-treated wild tomato root compared with that of control plant. In addition, two novel miRNAs were identified in the hypoxia-treated and untreated wild tomato roots as well, which shared the same sequence but generated from the different loci of the genome.

### Differentially expressed miRNAs related to auxin homeostasis may contribute to the phenotype of hypoxia-treated wild tomato roots

Previous studies showed that miR160, miR164, miR167, miR171, miR390, and miR393 were conserved with other species and involved in auxin homeostasis and signal transduction [12, 30–34]. In this study, miR164, miR167, and miR390 were observed to be expressed lower, whereas miR160 was expressed higher in hypoxia-treated wild tomato roots compared with untreated control plants (Figs. 3 and 4). However, there is little information about the function of these differentially expressed auxin homeostasis related miRNAs in tomato. To find the links between these miRNAs and the phenotypes present in hypoxia-treated wild tomato roots, the evidences reported in Arabidopsis and other species were used to explain the roles of these miRNAs regulating wild tomato root development. In Arabidopsis, higher expression of miR160 resulted in uncontrolled cell divisions and blocked cell differentiation in the root distal region, which subsequently generated a tumor-like root apex with loss of gravity-sensing by repressing its targets, *ARF10* and *ARF16* [32]. miR164a and miR164b double mutant plants expressed less miR164 and more *NAC1* mRNA and produced more lateral roots compared with WT plants, in Arabidopsis [30]. *IAR3*, is one of the target of miR167 in Arabidopsis, and fewer lateral root were present in *iar3* mutant plants, especially under high osmotic stress conditions [33]. Mutant of miR171 target gene, *SCL6,* exhibited decreased primary root elongation in Arabidopsis [35], and over expression of the target, *SlGRAS24,* strongly decreased the primary and lateral root growth compared with WT plants in tomato [12]. Combined with the fact that more and longer lateral roots were observed in STTM171 Arabidopsis compared with WT plants in our study (Fig. 7), demonstrating that miR171 have roles in regulating root development, including primary and lateral root, but the precise regulatory mechanism should be studied further. miR390, targets trans-acting short-interfering RNAs, *TAS3a*. It has been reported that *TAS3a* positively controls the lateral roots elongation in Arabidopsis [31]. Consistent with the phenotype exhibited in *TAS3a* mutant, more number and longer lateral roots were present in STTM390 transgenic Arabidopsis lines compared with WT plants (Fig. 8). Furthermore, auxin content was higher in hypoxia-treated wild tomato root compared with control plant (Fig. 1g), which is consistent with the fact that higher auxin content promote the formation of the lateral roots in rice [36]. All these evidences indicate that more and shorter roots of hypoxia-treated wild tomato at least partly result from differential auxin homeostasis related miRNAs. In addition, ethylene is a major hormone that interacts with auxin to regulate the root development in tomato [3, 37]. How these differentially expressed auxin homeostasis related miRNAs crosstalk with ethylene to give rise to more number and shorter roots in hypoxia-treated wild tomato should be studied further.

### Morphology maintenance-related miRNAs are differentially expressed between hypoxia-treated and control wild tomato roots

In this study, smaller plants with more and shorter root were found in hypoxia-treated wild tomato (Fig. 1), and miRNAs, such as miR159, miR171, and miR396, were lowly expressed in hypoxia-treated wild tomato root (Figs. 3 and 4). miR159 is highly conserved between Arabidopsis and tomato, *mir159ab* double mutant has multiple morphological defects, including reduced height, curled leaves, shorter siliques, and smaller and irregularly shaped seeds in Arabidopsis [38]. Similar phenotype of miR159 knockdown were also observed in STTM159 transgenic rice lines, such as dwarf plant, curled leaves, and small and irregularly shaped seeds [14]. Larger sized plants were observed when tomato overexpressed miR171, by virtue of repressing its target *SlGRAS24*. Consistent with this, dwarf plants were formed when *SlGRAS24* was overexpressed in transgenic tomato lines [12]. miR396, a conserved miRNA between Arabidopsis and tomato, has been recognized as a key regulator in cell proliferation in Arabidopsis, and plant height was reduced in miR396b overexpressing transgenic lines [39]. In tomato, shorter cotyledon length was shown in STTM396 transgenic lines compared with that of WT plant, whereas the flowers, sepals, and fruits of STTM396 transgenic plants were all significantly larger than those of WT plant [40]. Auxin homeostasis related miRNAs, such as miR160, miR164, miR167, and miR390, are also involved in plant root development through control of auxin homeostasis [41]. The above results indicate that the altered morphological changes of hypoxia-treated wild tomato may result from differentially expressed auxin homeostasis related miRNAs and miR159, miR171, and miR396.

### Stress-response related miRNAs are differentially expressed between hypoxia-treated and control wild tomato roots

Generally, plants show altered morphology when exposed to abiotic stresses. Dozens of miRNAs have been proven to change their expression profiles in response to various stresses to adapt the fickle environments [11]. miR169, a conserved miRNA family between Arabidopsis and tomato, has been recognized as a key regulator in response to abiotic stress [42, 43]. Previously, miR169l was reported to decrease in response to 1–3 days of waterlogging in the crown roots of maize seedling [18], which was consistent with the lower expression of miR169 in 12 days of hypoxia-treated wild tomato root (Figs. 3 and 4). Furthermore, Sorin et al. [44] reported that plants with reduced expression of miR169d,e,f,g exhibited shorter primary and lateral roots, whereas higher lateral root density were observed in plants overexpressing miR169 target, *NF-YA2,* when compared with WT Arabidopsis plants. This is also consistent with lower expression of miR169 in hypoxia-treated wild tomato root, which form more and shorter roots compared to the control plants (Fig. 1). Two other miRNAs, miR166 and miR482, target *HD-ZIP III* family transcriptional factors and genes coding for NBS-LRR proteins, respectively. Accumulated evidences have proven to show that these two miRNAs have roles in response to abiotic and biotic stresses [45, 46]. In addition, lower expression of miR166 and miR482 in hypoxia-treated wild tomato root (Figs. 3 and 4), indicated that miR166 and miR482 may also have roles in response to hypoxia treatment.

## Conclusions

In summary, our results combined with the data reported by other groups suggested that differentially expressed miRNAs, especially the miRNAs involved in regulating auxin homeostasis, root development, and stress response between hypoxia-treated and untreated wild tomato roots, may at least partially contribute to the smaller plants with more and shorter roots in hypoxia-treated wild tomato. Further studies will focus on the roles of the differentially expressed miRNAs, such as auxin homeostasis related miRNAs, to detect the molecular mechanism of these miRNAs in response to hypoxia in wild tomato.

## Additional files

Additional file 1: The primers used in this study. (XLSX 11 kb)

Additional file 2: Phenotype of 41 days old cultivar tomato plant at root rhizosphere under 12 days hypoxia treatment and control condition. (DOCX 176 kb)

Additional file 3: The miRNAs and their expression detected in hypoxiated and control wild tomato root by high-throughput sequencing technique. (XLSX 14 kb)

Additional file 4: The differentially expressed miRNAs between hypoxiated and control wild tomato root detected by high-throughput sequencing technique. (XLSX 13 kb)

Additional file 5: The expression of differentially expressed miRNAs verified by stem-loop qRT-PCR. Actin served as internal control. (DOCX 147 kb)

Additional file 6: Predicated targets of differentially expressed known miRNAs between hypoxiated and control wild tomato root by psRNATarget. (XLSX 84 kb)

Additional file 7: Validated targets of differentially expressed known miRNAs between hypoxiated and control wild tomato root by DPMIND. (XLSX 23 kb)

Additional file 8: The expression of differentially expressed miRNA targets detected by qRT-PCR. Actin served as internal control. (DOCX 99 kb)

Additional file 9: Predicted targets of novel miRNAs identified in this study by psRNATarget. (XLSX 11 kb)

Additional file 10: Comparison of lateral root number and length of Arabidopsis STTM171, STTM390 transgenic lines and wild type (WT). (DOCX 85 kb)

## Abbreviations

APS: ATP sulfurylase genes
ARF: Auxin response factor
GRAS24: GRAS transcription factor 24
HD-ZIP: HD-ZIP family transcription factors
IAR3: AA-Ala Resistant 3
NAC1: NAM/ATAF/CUC domain transcription factor 1
NBS-LRR: Nucleotide binding site-leucine-rich repeat
NFYA: Nuclear Factor Y, subunit A
PHO2: Phosphate over accumulator 2
PIN: PIN-FORMED gene
qRT-PCR: Quantitative real-time polymerase chain reaction
RDD1: Dof daily fluctuations 1
STTM: Short Tandem Target Mimic
SULTR2;1: Sulfate transporters 2;1
TAS3: Trans-acting short-interfering RNA 3
TPM: Transcripts per million
WT: Wild type
