# Supplementary material for: Identification and analysis of oxygen responsive microRNAs in the root of wild tomato (S. habrochaites)
Source: BMC Plant Biol. 2019 Mar 12;19:100. doi: 10.1186/s12870-019-1698-x (PMC6416974; doi:10.1186/s12870-019-1698-x)
Supplement: Supplementary file 2 — Phenotype of 41 days old cultivar tomato plant at root rhizosphere under 12 days hypoxia treatment and control condition. (DOCX 176 kb) [file 12870_2019_1698_MOESM2_ESM.docx]

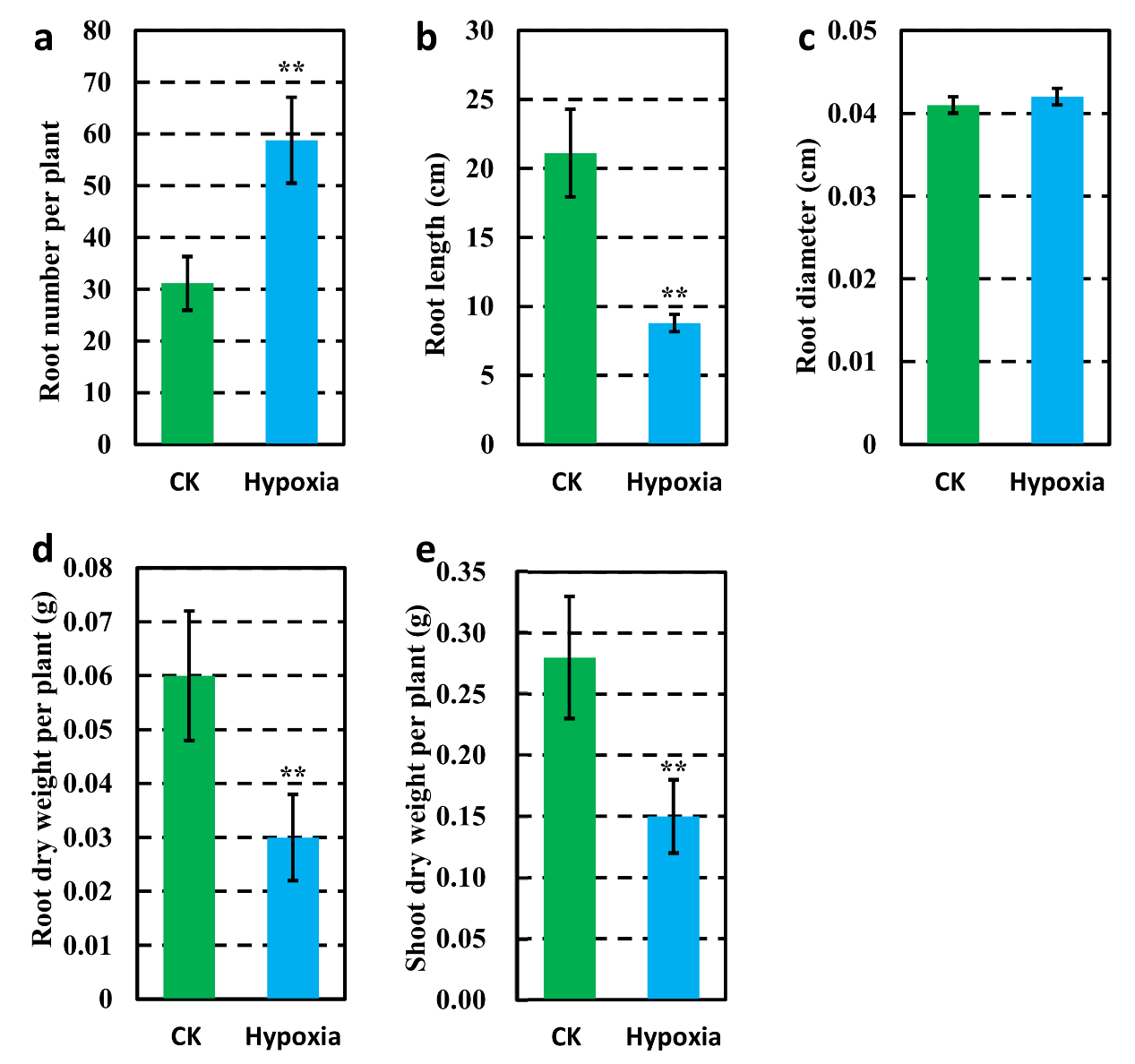


**Additional file 2. Phenotype of 41 days old cultivar tomato plant at root rhizosphere under 12 days hypoxia treatment and control condition.** (**a-e**) Statistical analysis of root number (**a**), root length (**b**), root diameter (**c**), dry weight of root (**d**), and dry weight of shoot (**e**). Asterisks indicate statistically significant differences compared with control by Student’s t test (*P < 0.05; **P <0.01).
