# Supplementary material for: Identification and analysis of oxygen responsive microRNAs in the root of wild tomato (S. habrochaites)
Source: BMC Plant Biol. 2019 Mar 12;19:100. doi: 10.1186/s12870-019-1698-x (PMC6416974; doi:10.1186/s12870-019-1698-x)
Supplement: Supplementary file 8 — The expression of differentially expressed miRNA targets detected by qRT-PCR. Actin served as internal control. (DOCX 99 kb) [file 12870_2019_1698_MOESM8_ESM.docx]

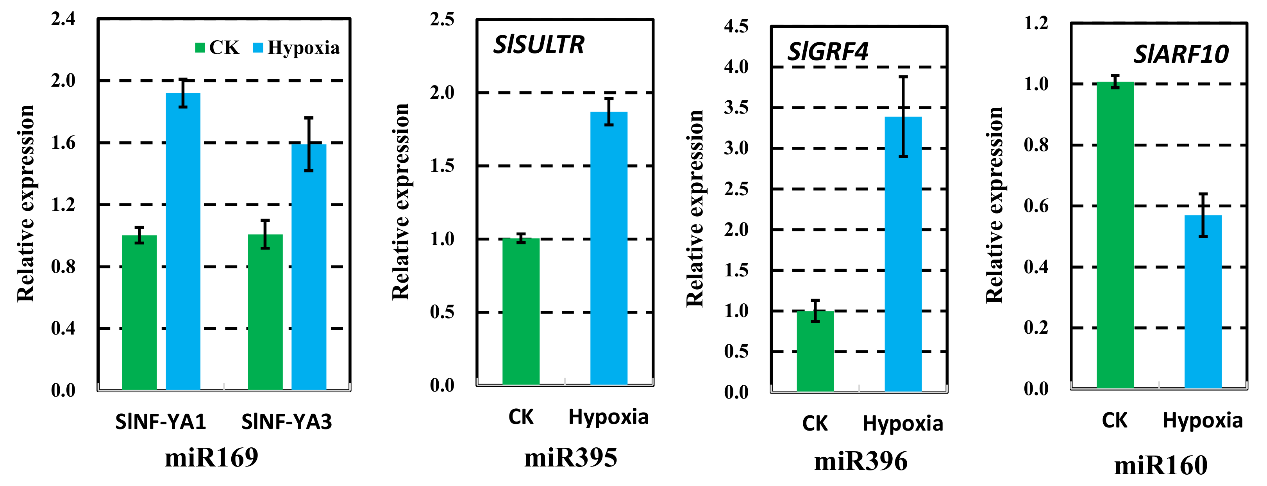


**Additional file 8. The expression of differentially expressed miRNA targets detected by qRT-PCR.** Actin served as internal control. Asterisks indicate statistically significant differences compared with control by Student’s t test (*P < 0.05; **P <0.01).
