# Supplementary material for: Identification and analysis of oxygen responsive microRNAs in the root of wild tomato (S. habrochaites)
Source: BMC Plant Biol. 2019 Mar 12;19:100. doi: 10.1186/s12870-019-1698-x (PMC6416974; doi:10.1186/s12870-019-1698-x)
Supplement: Supplementary file 10 — Comparison of lateral root number and length of Arabidopsis STTM171, STTM390 transgenic lines and wild type (WT). (DOCX 85 kb) [file 12870_2019_1698_MOESM10_ESM.docx]

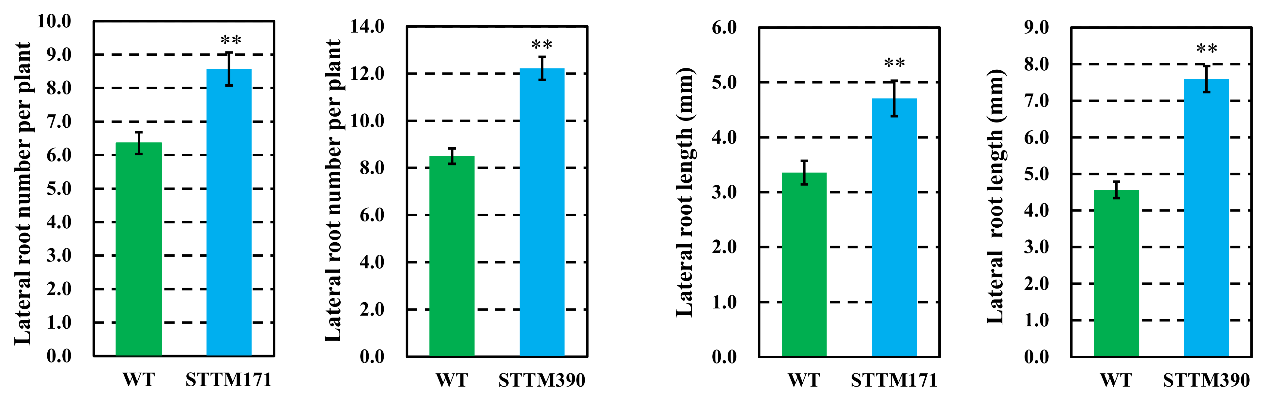
**Additional file 10. Comparison of lateral root number and length of STTM171, STTM390 transgenic lines and wild type (WT).** Asterisks indicate statistically significant differences compared with WT by Student’s t test (*P < 0.05; **P <0.01).
